# Different intrauterine environments and children motor development in the first 6 months of life: a prospective longitudinal cohort

**DOI:** 10.1038/s41598-023-36626-y

**Published:** 2023-06-26

**Authors:** Andressa Costa Wiltgen, Nadia Cristina Valentini, Thiago Beltram Marcelino, Luciano Santos Pinto Guimarães, Clécio Homrich Da Silva, Juliana Rombaldi Bernardi, Marcelo Zubaran Goldani

**Affiliations:** 1 Programa de Pós-Graduação em Saúde da Criança e do Adolescente, Universidade Federal do Rio Grande do Sul (UFRGS), Ramiro Barcelos, 2400, Santa Cecília, Porto Alegre, RS 90035-903 Brazil; 2 Hospital de Clínicas de Porto Alegre (HCPA), Porto Alegre, RS Brazil; 3 Programa de Pós‐Graduação em Ciências do Movimento Humano, Universidade Federal do Rio Grande do Sul (UFRGS), Porto Alegre, RS Brazil; 4 Escola de Educação Física, Fisioterapia e Dança, Universidade Federal do Rio Grande do Sul (UFRGS), Porto Alegre, RS Brazil; 5 Faculdade de Medicina, Departamento de Pediatria, Universidade Federal do Rio Grande do Sul (UFRGS), Porto Alegre, RS Brazil; 6 Serviço de Pediatria, Hospital de Clínicas de Porto Alegre (HCPA), Porto Alegre, RS Brazil; 7 Faculdade de Medicina, Departamento de Nutrição, Universidade Federal do Rio Grande do Sul (UFRGS), Porto Alegre, RS Brazil; 8 Serviço de Nutrição e Dietética, Hospital de Clínicas de Porto Alegre (HCPA), Porto Alegre, RS Brazil

**Keywords:** Risk factors, Intrauterine growth, Paediatric research, Neurodevelopmental disorders

## Abstract

This prospective cohort longitudinal study examines the risk factors associated with different intrauterine environments and the influence of different intrauterine environments on children’s motor development at 3- and 6-months of life. Participants were 346 mother/newborn dyads enrolled in the first 24 to 48 h after delivery in public hospitals. Four groups with no concurrent condition composed the sample: mothers with a clinical diagnosis of diabetes, mothers with newborns small for gestational age due to idiopathic intrauterine growth restriction (IUGR), mothers who smoked tobacco during gestation, and a control group composed of mothers without clinical condition. Children were assessed at three- and six-months regarding motor development, weight, length, head circumference, and parents completed a socioeconomic questionnaire. The IUGR children had lower supine, sitting, and overall gross motor scores at 6 months than the other children’s groups. Anthropometric and sociodemographic characteristics negatively influenced gross motor development. IUGR and anthropometric and sociodemographic characteristics negatively impact motor development. Intrauterine environment impact child neurodevelopment.

## Introduction

Inside the uterus, a child's neurological organization occurs on a continuum, from forming tissues in the central and peripheral nervous systems to the cortical activity's origin, initiating motor movements^1^. Humans’ first spontaneous and standardized fetal movements emerge around the seventh gestational week, alongside increased cortex activity^2^. After being born, the first months of the child's life are characterized by the activation of the spinal cord and brain stem's neural networks necessary for the predetermined motor repertoire. Then, the stimulation of permanent cortical circuits, favoring the adaptive process of movements, is related to the fast changes in gross motor development^2^. This phenomenon occurs around the child's fourth month, increasing its complexity throughout life^2^. This complex process is susceptible to environmental changes, and pre- and post-natal factors alternate in relevance regarding the impact on child development^3^.

Changes in the intrauterine environment can impair the mechanisms of placental transport, resulting in an inhospitable environment for the rapidly developing fetus’s growth^4^. Gestational Diabetes Mellitus (GDM) is considered one of the main intrauterine adverse environments, affecting approximately 14% of pregnant women worldwide^5^. Being overweight, obese, with micronutrient deficiency, having a family history of diabetes mellitus, and advanced age are well-known risk factors for this comorbidity^6^ Diabetes mellitus is the primary outcome of hyperglycemia, which causes morphological changes in the central nervous system's essential structures, and these are responsible for the balance and motor coordination of fetal systemic formation^7^. Embryonic and fetal oxidative stress is a mechanism that explains the embryotoxicity and teratogenicity caused by hyperglycemia^8–10^.

Consequently, the offspring are affected with adverse brain and behavior outcomes manifested during childhood, adulthood, or even as at old age. Children of women with GDM show motor^10–12^ and neurocognitive^13^ delays, and may have anatomical malformations, hypothalamic dysfunction, and neurodegenerative diseases^13^.

Another factor that can negatively impact children's development with a prolonged effect is exposure to tobacco during pregnancy^14^. Tobacco consumption during pregnancy can cause low birth weight, prematurity, and placental complications, leading to death^15^. Besides, intrauterine growth restriction (IUGR) affects about 5% of pregnancies worldwide. The most common and likely cause associated with idiopathic IUGR is uteroplacental insufficiency^16^. This gestational complication can directly impact the development of the central nervous system through impaired neurotransmission activity, decreased blood flow in the uteroplacental path, and uteroplacental insufficiency^16,17^; the effects may persist during the first year of life regarding poor postural control and low motor skills acquisition^18^.

A child's neurodevelopment is influenced by several factors, such as genetic inheritance^19,20^, disorders during pregnancy^21^, nutrition^3,22^, and families' socioeconomic status^23–25^. Low-income families have less access to information regarding children's health and development and healthy food^26^, live in neighborhoods lacking public safe spaces to be physically active^27,28^, and are more exposed to the consumption of tobacco^29,30^. The public healthcare system is fragile to support prenatal care, and low-income mothers are exposed to undetected prenatal risk^31^. Understanding the etiology of these adverse effects can help develop guidelines to promote children's development.

This prospective cohort longitudinal study examined the risk factors associated with different intrauterine environments and the influence of different intrauterine environments on children’s motor development at 3- and 6-months of life. Children exposed in the prenatal period to more risks and adverse intrauterine environment would demonstrate lower motor scores at 3- and 6-months of life.

## Results

### Intrauterine environment groups: risk factors comparisons

Several risk factors were associated with the IUGR group at birth, 3- and 6-months of age. Children in the IUGR group had lower weight (*p* < 0.001/*p* < 0.001), shorter length (*p* < 0.001/*p* = 0.005), lower head circumference (*p* < 0.001/*p* < . 012), and shorter length/age (*p* < 0.001/*p* < . 0.001), respectively. IUGR children also had lower weight/length (*p* < 0.001) and BMI/age (*p* < 0.001) at birth; and lower weight/age at birth (*p* < 0.001), 3-months (*p* < 0.001) and 6-months (*p* < 0.034) of age. Table 1 shows the children's risk factors by groups.

**Table 1:** Children risk factor by the intrauterine environments’ groups.

Children risk factors	Total (n = 346)	Intrauterine Environments Groups Comparisons				*p*
		DM (n = 62)	TOB (n = 87)	IUGR (n = 36)	CTL (n = 161)	
Child’s sex^1^						
Boys	160.0 (46.2)	29.0 (46.8)	44.0 (50.6)	15.0 (41.7)	72.0 (44.7)	.892
Girls	186.0 (53.8)	33.0 (53.2)	43.0 (49.4)	21.0 (58.3)	89.0 (55.3)	
Weight/Age^2^						
Birth	− .06 [− .86; .60]	.33 [− .24; 1.1]^a^	− .17 [− .96; .23]^b^	− 1.6 [− 1.9; − 1.4]^c^	.24 [.24; − .36]^a^	** < .001**
3 M	− .19 [− .80; .55]	− .07 [− .50; .80]^a^	− .38 [− 1.24; .33]^ab^	− .84 [− 1.3; − .21]^b^	.03 [− .05; − .64]^a^	** < .001**
6 M	.05 [− .62; .83]	.08 [− .40; 1.]^a^	− .33 [− .95; .62]^b^	− .35 [− 1.3; .49]^b^	.15 [.20; − .44]^a^	**.034**
Weight/Length^2^						
Birth	.37 [− .29; 1.1]	.91 [.21; 1.7]^a^	.34 [− .28; 1.1]^b^	− .56 [− 1.1; − .05]^c^	.48 [.41; − .20]^b^	** < .001**
3 M	− .05 [− .69; .79]	.16 [− .36; 1.0]	− .06 [− .65; .87]	− .34 [− 1.2; .59]	.05 [.02; − .77]	.371
6 M	.47 [− .49; 1.0]	.83 [− .15; 1.4]	.23 [− 0.49; 1.0]	− .21 [− .77; .81]	.23 [.54; − .48]	.163
BMI/Age^2^						
Birth	.07 [− .55; .94]	.82 [− .07; 1.4]^a^	.09 [− .47; .60]^b^	− 1.1 [− 1.6; − .76]^c^	.37 [.24; − .35]^ab^	** < .001**
3 M	− .17 [− .81; .55]	.01 [− .36; .72]	− .14 [− 1.02; .52]	− .61 [− 1.3; .17]	.00 [− .21; − .71]	.061
6 M	.33 [− .61; .97]	.71 [− .22; 1.3]	− .02 [− .67; .97]	− .21 [− .91; .69]	.14 [.40; − 0,64]	.156
Length / Age^**2**^						
Birth	− .56 [− 1.3; .37]	− .30 [− 1.2; .40]^ab^	− .71 [− 1.6; − .03]^a^	− 1.9 [− 2.4; − 1.3]^c^	− .19 [− .17; − .95]^b^	** < .001**
3 M	− .12 [− .98; .63]	.02 [− .74; .69]^a^	− .31 [− 1.2; .49] ^ab^	− .86 [− 1.4; − .11] ^b^	.06 [.09; − .63] ^a^	**.001**
6 M	− .22 [− .87; .68]	− .30 [− 1.0; .31]	− .49 [− 1.2; .52]	− .22 [− 1.3; .34]	.09 [.16; − .68]	.050
Weight (Kg)^2^						
Birth	3.2 [3.0; 3.5]	3.4 [3.2; 3.8]^a^	3.1 [2.8; 3.4]^a^	2.5 [2.4; 2.7]^b^	3.4 [3.1; 3.6]^a^	** < .001**
3 M	6.0 [5.5; 6.5]	6.3 [5.8; 6.7]^a^	6.0 [5.3; 6.4]^ab^	5.5 [5.2; 5.9]^b^	6.0 [5.7; 6.6]^a^	** < .001**
6 M	7.8 [7.1; 8.5]	8.0 [7.3; 8.4]	7.5 [6.9; 8.2]	7.3 [6.4; 8.5]	7.9 [7.2; 8.6]	.063
Length (cm)^2^						
Birth	49.0 [47.0; 50.0]	49.0 [47.0; 51.0]^ac^	48.3 [46.5; 50.0]^a^	46.0 [45.0; 47.0]^b^	49.0 [48.0; 50.5]^c^	** < .001**
3 M	60.5 [59.0; 62.1]	60.5 [59.1; 62.1]^a^	60.3 [58.5; 62.0]^ab^	59.0 [57.0; 60.4]^b^	60.9 [59.0; 62.5]^a^	**.005**
6 M	67.0 [65.2; 68.5]	67.0 [65.5; 68.0]	66.5 [64.5; 68.0]	65.7 [64.5; 68.0]	67.5 [66.0; 69.3]	.052
Head circumference (cm)^2^						
Birth	34.0 [33.0; 35.0]	34.0 [33.0; 35.0]^a^	33.5 [33.0; 34.5]^a^	32.0 [31.0; 33.0]^b^	34.0 [33.0; 35.0]^a^	** < .001**
3 M	40.0 [42.0; 44.0]	40.0 [39.5; 41.0]^a^	40.0 [39.0; 41.0]^a^	40.0 [38.7; 41.0]^b^	40.4 [39.1; 40.6]^a^	**.012**
6 M	43.1 [42.7; 44.0]	43.5 [42.6; 44.0]	43.0 [41.5; 43.9]	43.0 [41.0; 44.2]	43.2 [41.0; 44.2]	.387

^1^Qui-square (total sample with %); ^2^Kruskal-Wallis (median with quartiles 25; 75); Different letters represent different geometric values—Dunn's test. Analysis of standardized residue adjusted greater than or equal to 1.96. *p* < 0.05.

Significant values are in bold.

Several risk factors were more frequent for the TOB group of mothers than other groups. Smoking mothers had significantly lower formal education (*p* = 0.002), higher prevalence of living without a partner (*p* = 0.007), unplanned pregnancy (*p* = 0.001), and lower prenatal number of visits (*p* < 0.001) and SES (*p* = 0.001); these mothers also had a higher prevalence of vaginal delivery (*p* = 0.001). Maternal age was higher for the DM group (*p* < 0.001). Table 2 shows maternal-perinatal risk factors by groups.

**Table 2:** Maternal perinatal risk factors by intrauterine environments’ groups.

Maternal risk factors	Total (n = 346)	Intrauterine environment comparisons				*p*
		DM (n = 62)	TOB (n = 87)	IUGR (n = 36)	CTL (n = 161)	
Age (years)^1^	25.0 [21.0; 31.0]	29.0 [23.0; 33.0]^a^	23.0 [20.0; 29.0]^b^	23.5 [20.0; 27.5]^b^	25.0 [20.0; 31.0]^b^	** < .001**
Formal education (years)^1^	10.0 [8.0; 11.0]	10.0 [8.0; 11.0]^ab^	8.0 [7.0; 11.0]^c^	10.5 [8.0; 11.0]^ac^	11.0 [8.0; 11.0]^ab^	**.002**
Prenatal visits (numbers)^1^	8.0 [6.0; 10.0]	10.0 [8.0; 11.0]^a^	6.0 [4.0; 8.0]^c^	8.0 [5.0; 10.0]^cb^	8.0 [6.0; 10.0]^b^	** < .001**
Marital status^2^						
With partner	296.0 (79.6)	52.0 (83.9)	57.0 (65.5)	30.0 (83.3)	136.0 (84.5)*	**.007**
No partner	76.0 (20.4)	10.0 (16.1)	30.0 (34.5)*	6.0 (16.7)	25.0 (15.5)	
Ethnicity^2^						
White	223.0 (59.9)	40.0 (65.6)	51.0 (58.6)	16.0 (44.4)	98.0 (60.9)	.253
Non-white	148.0 (39.8)	21.0 (34.4)	36.0 (41.4)	20.0 (55.6)	63.0 (39.1)	
Planned pregnancy^2^						
Yes	130.0 (34.9)	29.0 (46.8)	14.0 (16.1)	14.0 (38.9)	63.0 (39.1)	**.001**
No	242.0 (65.1)	33.0 (53.2)	73.0 (83.9)*	22.0 (61.1)	98.0 (60.9)	
Type of delivery^2^						
Cesarean	126.0 (33.9)	28.0 (45.2)	22.0 (25.3)	12.0 (33.3)	47.0 (29.2)	**.001**
Vaginal	246.0 (66.1)	34.0 (54.8)	65.0 (74.7)*	24.0 (66.7)	114.0 (70.8)	
Breastfed 3M^2^						
Yes	214.0 (85.6)	37.0 (86.0)	48.0 (82.8)	22.0 (88.0)	92.0 (86.0)	.962
No	36.0 (14.4)	6.0 (14.0)	10.0 (17.2)	3.0 (12.0)	15.0 (14.0)	
Breastfed 6M^2^						
Yes	149.0 (69.0)	28.0 (73.7)	27.0 (62.8)	16.0 (66.7)	65.0 (69.1)	.796
No	67.0 (31.0)	10.0 (26.3)	16.0 (37.2)	8.0 (33.3)	29.0 (30.9)	
SES^2^						
A2 + B1	24.0 (6.5)	3.0 (4.9)	5.0 (6.0)	0 (0)	13.0 (8.4)	**.048**
B2	119.0 (32.1)	27.0 (44.3)*	17.0 (20.2)	12.0 (33.3)	50.0 (32.2)	
C1 + C2	204.0 (55.0)	31.0 (50.8)	54.0 (64.3)	19.0 (52.8)	84.0 (54.2)	
D + E	24.0 (6.5)	0 (0)	8.0 (9.5)*	5.0 (13.9)	8.0 (5.2)	

^1^Kruskal-Wallis (median with quartiles 25; 75); ^2^Qui-square (total sample with %); Different letters represent different geometric values—Dunn's test. Analysis of standardized residue adjusted greater than or equal to 1.96. p < 0.05.

Significant values are in bold.

### Difference between intrauterine environment groups in motor development

There were no significant differences in children's motor development by groups at 3-months of age. However, at 6-months, significant differences were found for supine, sitting, and gross motor skills. Children in the IUGR group had lower scores on the supine subscale than the children in the TOB group (*p* = 0.012); no other differences were found. Children in IUGR and DM groups had lower scores on the sitting subscale than the children in the TOB group, and children in the IUGR group had lower scores than those in the CTL group (*p* = 0.043). A similar trend was observed for the gross motor skills total score; the IURG had lower scores than children in the TOB and CTL groups (*p* = 0.037).

Overall, delays were low across all groups at 3-months of age, from no cases (DM groups) to 12% (IUGR); the IUGR group had a higher prevalence of delays than the other groups, but the differences were non-significant. At 6-months the prevalence of delays increased, across nearly 30% for DM, TOB and CTL groups. However, the IUGR group's prevalence of delays increased from 12 to 52%. This group had a significantly higher prevalence than the other three groups (*p* = 0.015). However, after adjusting the model for the type of delivery, children's sex, and maternal education, this significant result was no longer significant (*p* = 0.089). Table 3 shows the motor scores and categorization by groups at 3- and 6-months of age.

**Table 3:** AIMS results by intrauterine environments’ groups at 3- and 6-months of age: Non-adjusted and adjusted results.

AIMS scores & categorization	DM	TOB	IUGR	CTL	*p*	Adjusted p*
Follow-up: 3-months old	(n = 39)	(n = 53)	(n = 24)	(n = 96)		
Prone^1^	3 [2; 4]	3 [3; 5]	3 [2; 4]	3 [2; 4]	.222	.289
Supine^1^	4 [3; 4]	4 [3; 4]	4 [3; 4]	3 [3; 4]	.142	.119
Sitting^1^	2 [1; 3]^bc^	1 [1; 2]^a^	1 [1; 2]^ac^	2 [1; 3]^bc^	.046	.234
Standing ^1^	2 [2; 2]	2 [2; 2]	2 [2; 2]	2 [2; 2]	.672	.644
Gross Motor Skills^1^	11 [9; 13]	10 [8; 12]	10 [8; 12]	10 [8; 12]	.321	.232
Categorization^2^ n (%)						
Delay	0 (0.0)	2 (3.8)	3 (12.5)	4 (4.2)	.362	.535
Suspect	18 (46.2)	25 (47.2)	12 (50.0)	48 (50.0)		
Typical	21 (53.8)	26 (49.1)	9 (37.5)	44 (45.8)		
Follow-up: 6-months old	(n = 33)	(n = 35)	(n = 21)	(n = 77)		
Prone^1^	7 [6; 9]	7 [6; 9]	7 [7; 8]	7 [6; 9]	.999	.987
Supine^1^	5 [4; 7]^ac^	7 [5; 8]^bc^	5 [4; 6]^a^	6 [4; 7]^ab^	**.008**	**.012**
Sitting^1^	4 [2.5; 6]^ac^	5 [4; 7]^b^	3 [2; 5]^a^	5 [3; 6]^bc^	**.022**	**.043**
Standing^1^	3 [2; 3]	3 [2; 3]	3 [2; 3]	3 [2; 3]	.379	.274
Gross Motor Skills^1^	20 [17; 21]^ac^	22 [18; 25]^a^	17 [16; 21]^bc^	20 [18; 24]^a^	**.046**	**.037**
Categorization^2^ n (%)						
Delay	11 (33.3)	7 (20.0)	11 (52.4)^#^	21 (27.3)	**.015**	.089
Suspect	21 (63.6)	18 (51.4)	9 (42.9)	38 (49.4)		
Typical	1 (3.0)	10 (28.6)	1 (4.8)	18 (23.4)		

^1^Kruskal-Wallis and ANCOVA (median with quartiles 25; 75). ^2^Qui-square (total sample %); *p value was adjusted for type of delivery, sex of the child, and maternal education. Different letters represent statistically different distribution—Dunn's test. Analysis of standardized residue adjusted greater than or equal to 1.96. *p* < 0.05.

Significant values are in bold.

**Figure 1:**
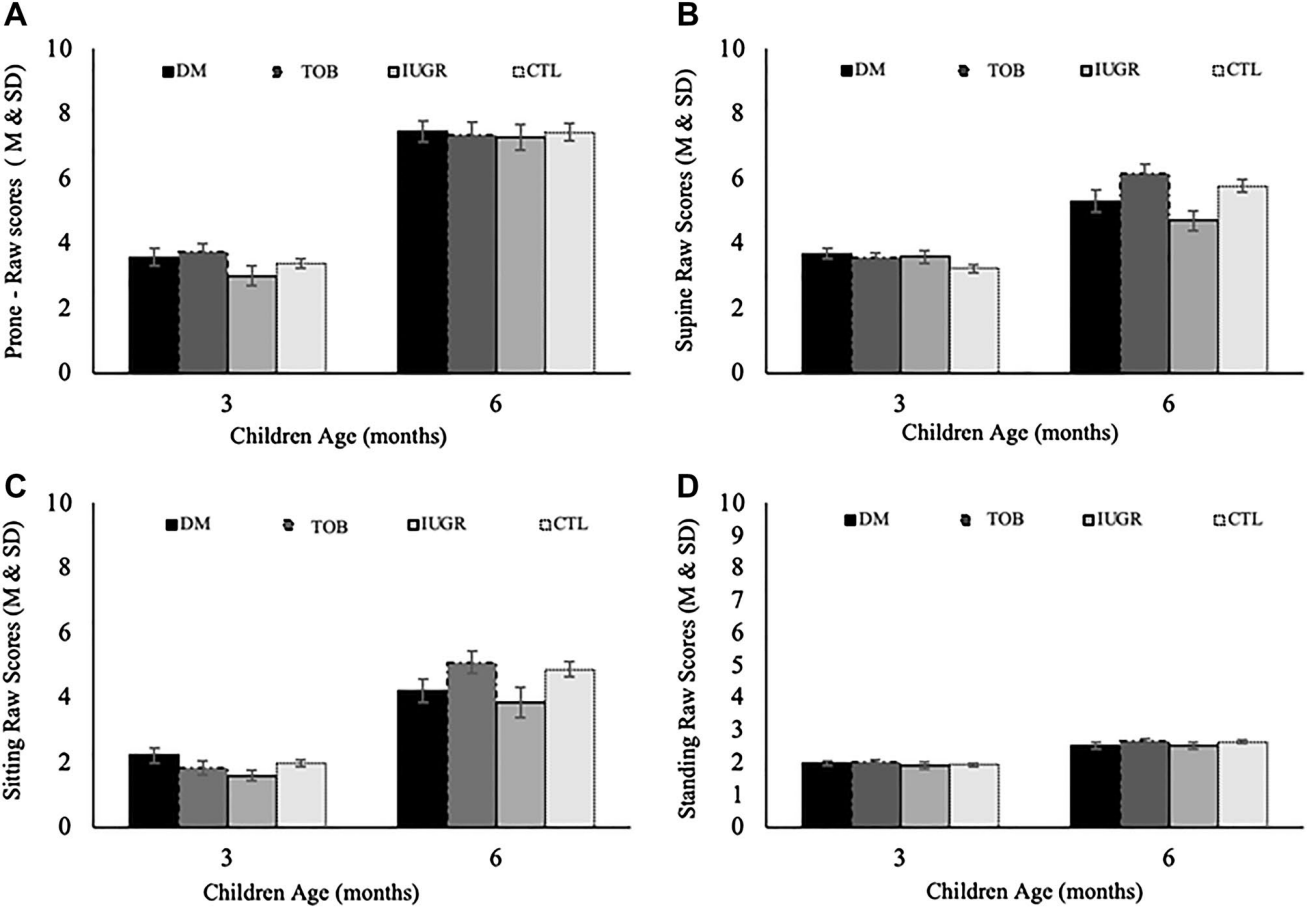
Raw scores mean and standard deviation by intrauterine environments groups at the prone, supine, siting and standing postures.

Figure 1 provided the mean scores and standard deviation by intrauterine environments groups at the prone, supine, sitting, and standing postures. Positive changes in motor performance were observed over time (3- to 6 months) for all groups, although changes were less evident for the children with IUGR.

Figure 1

Besides, yet regarding longitudinal changes, for the supine subscale, a significant group-by-time interaction was found (*p* = 0.005). The IUGR group increases in the scores were significantly lower than the other groups from 3 to 6 months. Table 4 shows GEE comparisons by time, groups, and time-by-groups (see also Supplementary S1 online).

**Table 4:** Generalized Estimating Equation model results: Groups, time, and Group by time interactions at 3- and 6-months.

AIMS	Groups	Longitudinal motor scores		*p*		
		3 months (n = 212)	6 months (n = 166)	Group	Time	Group*time
Prone	DM	3.6 [3.1; 4.1]	7.4 [6.8; 8.1]	.404	** < .001**	.468
	TOB	3.7 [3.3; 4.3]	7.4 [6.6; 8.1]			
	IUGR	3.0 [2.5; 3.7]	7.3 [6.5; 8.1]			
	CTL	3.4 [3.1; 3.7]	7.4 [6.9; 8.0]			
Supine	DM	3.7 [3.4; 4.0]^aA^	5.3 [4.7; 6.0]^abB^	.054	** < .001**	**.005**
	TOB	3.5 [3.2; 3.9]^aA^	6.2 [5.7; 6.7]^aB^			
	IUGR	3.6 [3.2; 4.0]^aA^	4.7 [4.1; 5.3]^bB^			
	CTL	3.2 [3.0; 3.5]^aA^	5.8 [5.4; 6.2]^aB^			
Sitting	DM	2.2 [1.8; 2.7]	4.2 [3.5; 4.9]	**.041**	** < .001**	.236
	TOB	1.8 [1.5; 2.3]	5.1 [4.5; 5.8]			
	IUGR	1.6 [1.3; 1.9]	3.8 [3.0; 4.9]			
	CTL	1.9 [1.8; 2.2]	4.9 [4.4; 5.3]			
Standing	DM	1.9 [1.8; 2.1]	2.5 [2.3; 2.7]	.470	** < .001**	.738
	TOB	2.0 [1.9; 2.1]	2.7 [2.5; 2.8]			
	IUGR	1.9 [1.7; 2.1]	2.5 [2.3; 2.7]			
	CTL	1.9 [1.8; 2.0]	2.6 [2.5; 2.7]			
Gross Motor Skills	DM	11.4 [10.4; 12.5]	19.2 [17.9; 20.6]	.09	** < .001**	.124
	TAB	10.9 [9.9; 12.1]	21.2 [19.8; 22.8]			
	IUGR	10,0 [8.9; 11.2]	18.3 [16.7; 20.1]			
	CTL	10.4 [9.9; 10.9]	20.7 [19.5; 21.8]			

It was adjusted for type of delivery, sex of the child and maternal education; Distinct lowercase letters represent differences by time compared with groups; Distinct capital letters represent different means and compared with time; *p* < 0.05.

Significant values are in bold.

Table 4

## Discussion

This cohort longitudinal study investigated the influence of different intrauterine environments on children's gross motor development in the first 6 months of life, an original contribution to the current knowledge. Several risk factors were associated with the IUGR group at birth, 3- and 6-months of age; those children had lower scores on all anthropometric measures, like previous studies^32,33^. For example, Shoji and colleagues^34^ reported that the IUGR affects birth weight, body length, and BMI of children during the first six years and that there was no catch-up in birth weight and BMI during these years with children were small for the gestational age. The present study supported these findings for Brazilian children in the first 6 months of life. Characterizing distinct trajectories of BMI in pediatric populations according to the intrauterine environment is an original contribution of the present study; this information may be helpful to improve the identification of relationships between growth and health outcomes among children and to develop healthcare strategies.

Besides, children with atypical motor development or suspected motor delays had higher risk factors at birth. More risk factors were associated with mothers that smoke and children with idiopathic intrauterine growth restriction; children in this group also had lower scores in supine and sitting posture and overall gross motor skills at 6-months. Children with lower weight and small length and head circumference at birth, regardless of intrauterine comorbidity, were the ones with more prevalence of motor development atypical or with delays in the first 6 months of life. Our results were aligned with previous studies that reported alterations in gross motor development, not only in the postnatal period but also throughout the first year of life^35–38^. This study provided evidence for the first 6 months of life, a period with several changes in brain development, such as dendritic refinement, myelination, and extensive reorganization of synapses. These neural modifications allow more complex movements since the muscle stimulus collects perceptual, cognitive, and social information^2,39^. Therefore, early diagnosis and intervention are critical to take advantage of this opportunity window and prevent future motor deviations^39^.

The present study also found that mothers that smoke had several other risk factors associated, such as lower formal education and SES, higher prevalence of living without a partner, unplanned pregnancy, and lower prenatal number of visits, and there were also more single mothers in this group, like previous studies^40,41^. For example, two cohort studies, one in Finland^40^ and another in Japan^41^, showed that pregnant women who smoke during pregnancy are from socioeconomically disadvantaged groups with low formal education^40,41^; such aspects may result in an unplanned pregnancy and a decrease in prenatal visits^40^, our results confirm this results for Brazilian mothers. The results worldwide, regardless of the differences in countries’ economies—Japan and Finland are categorized as high-income developed economies, whereas Brazil is an upper middle-income developing economy^42^—suggested the need to develop strategies to prevent smoking among pregnant women living in poverty.

Interestingly the mothers in the TOB group also had a higher prevalence of vaginal delivery. There is two possible complementary plausible explanation for this result. First, the groups in the present study were designed to be mutually exclusive. No mothers in the TOB group had a case of IUGR—cases that c-sections are more often required. Second, the TOB group was composed of socioeconomically disadvantaged mothers who were more likely to deliver their babies in public hospitals. In Brazil, no decline in the tendency for cesarean delivery has been observed; a similar trend has been reported worldwide^43^. However, there are disparities in cesarean rates when comparing the care performed in the Unified Health System—45% (SUS—public hospitals) and supplementary private health—90%^44^. The emerging demand for vaginal delivery in SUS and supplementary private health has been observed. Although this demand has not been met at an intensity that will positively affect the reduction of cesarean rates^43^, public hospitals seem more inclined to promote vaginal delivery^44^.

Regarding children, our group of children with idiopathic intrauterine growth restriction and were small for gestational age had high-risk factors (had lower weight, shorter length, lower head circumference, and shorter length and weight or their age) as well as lower scores in supine and sitting postures, and overall gross motor skills at 6-months, supporting our hypothesis. Similar results have been reported regarding low weight, short length, small head circumference^41,35^, and low motor scores^18,45,46^, which corroborates the results from our study. For example, a systematic review with 29 studies reported that children with fetal growth restriction and small for the gestational age had overall low neurodevelopmental scores (i.e., 0.32 standard deviation below control groups), although with variable effect sizes^46^.

We expected that children from the tobacco group would have higher risks and demonstrate low motor scores than the control group, but the hypotheses were not confirmed. Mothers that smoke had several risk factors; however, their children had similar characteristics and motor scores to the control group. Our results are not aligning with previous research^41,47^. For example, a cohort study realized in Japan reported that children of mothers who smoked during pregnancy were at higher risk of low birth weight^41^. Another study suggested that pregnant women exposed to tobacco had children with a higher prevalence of motor development delays; however, it is crucial to notice that this study also suggested that the motor deficit was reversed in the following months of life^47^. One of the study weaknesses would be in the TOB group. During the grouping process, children with low birth weights were excluded from this group; the intrauterine environment groups were designed to be mutually exclusive. This study design criterion may explain the higher anthropometric parameters when compared TOB to the other groups. Usually, children exposed to tobacco during the intrauterine period had lower motor scores^15,48,49^, and we did not find this result in our study.

Gross motor milestones acquisition in children progresses parallel to the maturation of different regions of the central nervous system^1^. At 3 months, the child presents only pre-determined movements such as reflexes and sagittal plane movements (flexion and extension)^2,50,51^. From the fourth month of life, motor learning begins with the appearance of movement in the frontal plane (weight transfer)^2,51^ . When the child reaches 6 months, more prominent motor learning complexity provides a greater diversity of movements in the sagittal, frontal, and transverse planes (rotations)^2^. These functional neurophysiological characteristics of infant motor development may explain the more substantial number of movements found at 6-months compared to 3-months of age within the AIMS scale; improvements in motor performance over time were found for all groups.

The first strength of this study is its design, a baseline study for children's motor development regarding examining the longitudinal effects of four intrauterine environments in a cohort design, mothers with diabetes mellitus, who smoked, and newborns small for gestational age due to idiopathic intrauterine growth restriction, and a control group. Another strength of the present study was the sample inclusion criteria; several possible biases of sample composition was overcame with a precise group definitions, controlling for social factors and clinical effects of gestational age.

The study has several limitations. The lack of information regarding mothers' daily tobacco intake. Although we attempted to collect this information, mothers could not provide data precisely. Another limitation concern missing subjects in the follow-up assessments; this is a common difficulty when carrying out longitudinal studies, especially cohort studies. The missing subjects through the longitudinal assessments and the consequence of the unequal distribution of the groups were limitations of the present study that, despite the several strategies adopted, were not overcome by the research team. Overall, missing data was due to the socioeconomic and demographic characteristics of the sample. The mothers joining the workforce after childbirth, the family's informal work and consequent mobility to other cities to find better job opportunities, and the lack of fixed residence—families no longer lived in the same place where the initial interviews were conducted—increased the difficulties of contacting and re-engaged the families in the research.

## Conclusion

In conclusion, the possible deleterious effects of the intrauterine environments presented had a negative influence on neurological motor development at 6 months of life. It should be considered that delays in the child's motor development can negatively impact their opportunities to explore the environment and experiment with new motor challenges. This study can be used as a perspective for research that evaluates the contribution of the mother–child bond and environmental factors to the outcome of child motor development. Early diagnosis and intervention can positively influence maternal and child health, impacting Brazilian public health.

## Methods

### Study design and participants

This study is part of a prospective longitudinal cohort regarding the impact of perinatal different intrauterine environments on child growth and development in the first 6 months of life (IVAPSA)^52^. It was approved by the Human Research Ethics Committees of the Hospital de Clínicas de Porto Alegre (reference n° 110097) and Grupo Hospitalar Conceição (reference n° 11027). All the protocols were conducted in accordance with national and international health guidelines and regulations. Informed consent terms were applied to all participants or their legal guardians. The target population consisted of mothers and their newborns from two large public hospitals in Brazil. The mother/children’s dyads were recruited in the first 24 to 48 h after the child's birth. The exclusion criteria were HIV seropositive mothers, gestational age less than 37 weeks, twin pregnancy, newborns with congenital diseases, and newborns requiring hospitalization.

Hospital records were used to reach the mothers/newborns dyads target groups: diabetes mellitus (DM) group composed of mothers with a clinical diagnosis of diabetes, tobacco (TOB) group composed of mothers who smoked at any moment during the gestation, intrauterine growth restriction (IUGR) group composed of mothers with newborns small for gestational age due to idiopathic intrauterine growth restriction, and control (CTL) group composed of mothers without any known clinical condition. Groups were designed to be mutually exclusive, without any overlap between diagnoses. Therefore, children in the TOB group who also showed IUGR were removed from the sample. Similarly, no mother with diabetes also used tobacco in the sample.

Puerperal women were invited to participate in the research and signed the informed consent form before the investigation. Initially, 346 mother/newborn dyads participated in a postpartum interview, the study's first phase. At the study's follow-up, children were assessed at 3 months of age using the Alberta Infant Motor Scale (AIMS)^53,54^; 38% of the mother/children’s dyads discontinued the participation—212 children were assessed. At the second follow-up, at 6-months, children were reassessed using AIMS; a further discontinuity of participation occurred, and 166 mothers/children’s dyads were assessed.

Mothers complete a socioeconomic questionnaire (i.e., age in years, marital status, ethnicity, formal education, number of prenatal consultations, and delivery type. We use a national assessment to examine the socioeconomic status (SES)^55^. Family SES is organized into eight socioeconomic strata (A, A2, B1, B2, C1, C2, D, and E); families’ income and purchasing power define the categories^55^.

### Assessment and procedures

Children’s sex, weight (Kg), head circumference (cm), length at birth (cm), and gross motor development, using the Alberta Infant Motor Scale (AIMS)^53,54^, were used at the follow-ups at 3- and 6-months of age. The AIMS is a tool to assess gross motor skills, with 58 dichotomous items organized in four subscales (prone, supine, sitting, standing). The assessor observes the children's postural alignment, antigravity movements, and weight-bearing. The children’s assessments were conducted in a quiet room on a flat surface for 20 min. The AIMS provides raw motor gross scores, age percentiles, and categorization of motor development; validity and reliability have been established for Brazilian children^54,56^.

Weight (Kg) was measured on a flat surface, using a digital scale with a maximum 150 kg capacity and 50 g (g) accuracy. The weight was obtained with the mother in a vertical position, barefoot and wearing light clothes, afterward, with the child on her lap and subtracting the mother's weight to register the child's. The child's length was analyzed on a flat surface using a professional anthropometer in the supine position. All data were measured twice, and the average was recorded. Trained researchers conducted the assessments. We used the World Health Organization (WHO) *Anthro*® software. Children’s growth parameters were assessed according to anthropometric weight for age, weight for length, body mass index for age (BMI /Age), and length for age guidelines^57^.

### Data analysis

A Shapiro–Wilk normality test was performed to identify the distribution curve of the analyzed variables, thus defining which descriptive and inferential statistics would be used. Absolute numbers and percentages were used to represent categorical variables. Mean, standard deviation, median, and interquartile (range p25; p75), were used for the quantitative variables. Groups with sample sizes smaller than 12 participants were represented by the median and interquartile range and analyzed by non-parametric tests. Kruskal–Wallis test was used for asymmetric variables (including intrauterine groups compared to AIMS score) and, if significant, used Dunn's pair for *posthoc* tests. Different proportions were compared using Chi-square test for the between groups’ scores and classifications (delays, suspicious, and typical motor development). The AIMS scores were adjusted for the type of delivery, sex of the child, and maternal education using ANCOVA.

The generalized Estimation Equation (GEE) model was used to compare the motor changes longitudinally (3- and 6-months-old), evaluating the effects of time, group, and group by time. The logistic model in the GEE was used to examine the longitudinal data since it involves repeated measurements of scores that tend to correlate with one another, which must be taken into proper account. The GEE models allow for substantial flexibility in specifying the correlation structure within cases and offer the potential for valuable substantive insights into the nature of that correlation. The GEE also allowed for the intra-group comparison in each assessment period, preventing the bias of multiple independent comparisons^58,59^. This model comprised an unstructured working correlation matrix—a robust estimator covariance matrix using a normal distribution with identity linkage function, using Bonferroni's *posthoc* tests, if significant^60,61^. Statistical analyses were performed using the Statistical Package for the Social Sciences (SPSS) version 18.0, and *p* < 0.05 was adopted as the significance level.

## Supplementary Information

Supplementary Figure 1.

## Data Availability

The datasets generated and/or analyzed during the current study are not publicly available because they are part of a larger study, with analyses still being carried out. For this reason, the authors of this article have not released the data for public access on any online platform. If requested, the corresponding author can send them directly by email.
