# Supplementary material for: Different intrauterine environments and children motor development in the first 6 months of life: a prospective longitudinal cohort
Source: Sci Rep. 2023 Jun 26;13:10325. doi: 10.1038/s41598-023-36626-y (PMC10293270; doi:10.1038/s41598-023-36626-y)
Supplement: Supplementary file 1 — Supplementary Figure 1. [file 41598_2023_36626_MOESM1_ESM.docx]

**

**

**S1-** Figure representing the Generalized Estimating Equation model results, evaluating the evolution of different AIMS positions from the third to the sixth month of life of child from different intrauterine environments: A) Prone mean score; B) Supine mean score; C) Sitting mean score; D) Standing mean score. Note: The different intrauterine groups are represented by different colors. It was adjusted for type of delivery, sex of the child and maternal education; *p<0.05.
